# Reported concepts for the treatment modalities and pain management of temporomandibular disorders

**DOI:** 10.1186/s10194-015-0586-5

**Published:** 2015-12-07

**Authors:** Mieszko Wieckiewicz, Klaus Boening, Piotr Wiland, Yuh-Yuan Shiau, Anna Paradowska-Stolarz

**Affiliations:** Department of Prosthetic Dentistry, Faculty of Dentistry, Wroclaw Medical University, 26 Krakowska St., 50425 Wroclaw, Poland; Department of Prosthetic Dentistry, Faculty of Medicine, Dresden University of Technology, Fetscherstrasse 74, 01307 Dresden, Germany; Department and Clinic of Rheumatology and Internal Medicine, Faculty of Medicine, Wroclaw Medical University, 213 Borowska St., 50556 Wroclaw, Poland; Department of Prosthetic Dentistry, School of Dentistry, National Taiwan University, 1 Changde St., 100 Taipei City, Taiwan; Department of Maxillofacial Orthopedics and Orthodontics, Faculty of Dentistry, Wroclaw Medical University, 26 Krakowska St., 50425 Wroclaw, Poland

**Keywords:** Temporomandibular disorders, Temporomandibular joint disorders, Facial pain, Masticatory muscle pain

## Abstract

**Background:**

Pain related to temporomandibular disorders (TMD) is a common problem in modern societies. The aim of the article is to present the concepts of TMD pain clinical management.

**Methods:**

A survey was performed using the PubMed, SCOPUS and CINAHL databases for documents published between 1994 and 2014. The following search keywords were selected using MeSH terms of the National Library of Medicine in combination: TMD pain, TMD, TMJ, TMJ disorders, occlusal splint, TMD physiotherapy, TMJ rheumatoid disorders and TMJ surgery. Original articles and review papers which presented the clinical relevance and practical validity regarding the possibility of application in TMD management have been included. Authors have excluded articles without outstanding practical aspect and evidence-based background. A first selection was carried out by reviewing titles and abstracts of all articles found according to the criteria. After that the full texts of potentially suitable articles were assessed. In line with these criteria, among 11467 results the writers have included 66 papers.

**Results:**

The most commonly reported conservative treatments are massage therapy and individually fabricated occlusal splints. In addition to massage, other popular methods include manual therapy and taping, warming/cooling of aching joints, and light and laser therapy. Drugs are also commonly used. In the most severe cases of the temporomandibular joint degeneration, surgical restoration of the joint is sometimes applied.

**Conclusions:**

The authors concluded that conservative treatment including counselling, exercises, occlusal splint therapy, massage, manual therapy and others should be considered as a first choice therapy for TMD pain because of their low risk of side effects. In the case of severe acute pain or chronic pain resulting from serious disorders, inflammation and/or degeneration pharmacotherapy, minimally invasive and invasive procedures should be considered.

## Introduction

Currently, temporomandibular disorders (TMD) refer to the causes responsible for the impaired function of the temporomandibular joints (TMJ) and the associated neuro-muscular system, which may provoke TMD-related pain [1]. The term TMD is not a diagnosis but rather a broad term that contains a number of disease entities, such as pain in masticatory muscles and temporomandibular joints, headache, disturbances in jaw movements and sounds in joints while opening and closing the mouth. The causes of these diseases/symptoms are numerous and include trauma, systemic, iatrogenic, occlusal and mental health disorders [2–7]. Today, mental health plays a dominating role in the pathogenesis of TMD [8, 9]. The neuromuscular system responsible for chewing function has a high potential to adapt to changing conditions. Only when the compensatory capabilities of the masticatory- and the neuromuscular system are overstretched dysfunction occurs resulting in clinical symptoms and manifests as pain, severe clicking, or limited mobility of the mandible, forcing the patient to seek help.

The pain may radiate to different regions, such as the dental arches, ears, temples, forehead, occiput, cervical region of spine or shoulder girdle [10–13]. However, despite the fact that comparatively few patients are seeking treatment, it is known that there is a high prevalence of TMD in developed societies [14, 15]. TMD is mostly accentuated on the neck, where the lateral support imbalance leads to the bending of the neck to the affected side [16].

TMD are a group of dysfunctions and disorders related to impaired function of the temporomandibular joints and associated muscles therefore they may lead to the painful impairment in stomatognathic system functioning [17]. The TMJ is used 1500–2000 times a day, which shows how great discomfort is carried by the pathologies in jaw movements [9].

In most cases, the symptoms are the reason for the increased tension of the masticatory musculature, and the parafunctions may worsen the symptoms [18, 19]. Due to the large subjectiveness of the symptoms, TMDs are very difficult to diagnose, especially because patients usually search for help from other specialists besides dentists (e.g., neurologist, otolaryngologist or ophthalmologist) [10, 20]. The anomalies of the masticatory system including pain caused by increased tension of masticatory muscles are classified as masticatory pain dysfunction syndrome (MPDS) [21].

In addition to pain, a vast majority of patients suffer from intraoral signs of masticatory dysfunction, including increased sensitivity of the teeth due to abfraction and pathological attrition, gingival recessions, teeth hypermobility and bone support loss. In addition, teeth impressions on soft tissues are observed, including teeth impressions on the tongue and (cheek mucosa) *linea alba* [10, 22]. The increased tension in TMJ muscles and co-existing parafunctions or dysfunctions may lead to non-carious tooth lesions (e.g., abfraction), which are characteristic for TMD [23, 24].

The treatment of TMD is complicated and requires specific knowledge and exercises to strengthen some groups of muscles and weaken others, occlusal splint therapy, massage and pharmacotherapy. Although the treatment seems difficult, most of the patients searching for help due to TMD assess that the treatment is successful, although an accurate diagnosis needs to be made to start the proper protocol of treatment [20, 25–27]. Theories on the origin of TMD are presented in Table 1 [27]. Yet, it is important to note that treating TMD only from the dental perspective may fail, as many of these anomalies are caused by somatic diseases that should have be cured in the first place [28].

**Table 1:** Theories concerning TMD origin [27]

Name of the theory	Statements of the theory
Mechanical displacement (by Costen)	Lack of support in lateral teeth or functional occlusal premature contacts lead to direct eccentric positioning of the condyle in the glenoid fossa; this leads to pain, ear symptoms, adverse muscle activity and TMD
Trauma theory (by Zack and Speck)	The principal factor of TMD is micro-/macro-trauma; trauma can cause structural alternation to the muscles or directly to the joint structures
Biomedical (by Reade)	Disorder is initiated by trauma; specific factors (malocclusion, parafunctions, occupational activities) cause the progression of the symptoms
Osteoarthric (by Stegenga)	Osteoarthrosis is a main cause of TMD; muscular symptoms and systemic diseases are secondary to TMJ pathology
Muscle (by Travell and Rinzler)	Masticatory muscles are the primary etiologic factor to TMD; myalgia (caused by chronic myospasm) is secondary to parafunctions and can refer pain to TMJ
Neuromuscular (by Ramfjord)	Occlusal problems cause TMDs, the loss of occlusal equilibrium leads to the incoordination of muscles and spasms
Psychophysiological (by Schwartz and Laskin)	TMD occurs outside of the physical factors; psychosocial factors play a crucial role in TMD pathogenesis – the main factor of hypertension and overcontraction of the muscle is due to the parafunctions performed to relieve stress
Psychosocial theory (by Dworkin)	Emotional disturbances induce hyperactivity of the muscles and lead to parafunctional habits and occlusal anomalies; the muscle contractivity is accentuated with teeth clenching, and repeatability leads to pain

The prevalence of these disorders and the multifactorial pathogenesis and therapeutic difficulties of TMD prompted the authors to undertake an effort to describe therapeutic concepts associated with TMD pain.

## Review

### Materials and methods

A survey was performed using the PubMed, SCOPUS and CINAHL databases for documents published between 1994 and 2014. The following search keywords were selected using MeSH terms of the National Library of Medicine in combination: TMD pain, TMD, TMJ, TMJ disorders, occlusal splint, TMD physiotherapy, TMJ rheumatoid disorders and TMJ surgery. Original articles and review papers which presented the clinical relevance and practical validity regarding the possibility of application in TMD management have been included. The inclusion of the papers were based on precise descriptions of the treatment procedures and detailed presentation of the treatment outcomes. Authors have excluded articles without outstanding practical aspect and evidence-based background. A first selection was carried out by reviewing titles and abstracts of all articles found according to the criteria. After that the full texts of potentially suitable articles were assessed. In line with these criteria, among 11467 results the writers have included 66 papers.

## Conservative treatment

### Therapeutic exercises

The most important stage of a treatment protocol is education with cognitive awareness training and relaxation therapy as well as self-observation that should be completed by patients with masseter hypertrophy, tension-type headaches or bruxomania (the grinding of teeth occurring as a neurotic habit during the waking state). It is important to explain to the patient the background of the disorders (especially the role of one’s emotional stress) and warn about habitual parafunctional activities (e.g., nonfunctional tooth contacts or oral mucosa biting). The patient should be aware of what he or she does with their teeth, and when they fall into bad habits, try to eliminate that habit [28].

Muscular training is the primary mode to achieve muscle restoration, especially after traumas and injuries. It is thought to be the most conservative treatment as well as the simplest and most non-invasive method of TMD treatment. In patients with severely expressed asymmetries and symptoms, exercises to restore the muscular equilibrium seem to be the only proper route of treatment [3, 29]. Muscular therapy must be restrictive; it should be carried out moderately, and the intensity should be increased with time to avoid aches and patient discouragement from the suggested treatment. In this situation, muscular therapy is effective in 70 % of suffering patients. In some cases, such as patients with muscular or joint (muscular or arthritis pain) pain, the mouth opening is limited, and therefore, therapy is less effective [3, 27]. The exercises can require stretching, relaxation and isometric movements that should be performed routinely to eventually lead to a shortening of the excessively expanded muscles or to a restoration of the full length of the shortened muscles. Additionally, the natural tension and symmetric jaw movement can be restored [3].

The training is underdone to correct the mobility of the mandible. To strengthen the muscles and to acquire balance between the left and right sides, opening the mouth along a straight line in front of the mirror is recommended. The resistance is acquired from the gentle pressure of the patient’s fingers to the mandible. The exercises are repeated in sets of 15 to 20 repetitions, 2 to 3 times a day. The improvement should be observed after 6 weeks [3].

Research from Bae and Park [30] showed that active and relaxation exercises could improve the limited range of motion, deviation and pain in masticatory muscles. For muscle relaxation, they recommend putting the front one-third of the tongue on the anterior part of palate and applying a light force to the tip of the tongue so it does not touch the teeth, having the patient maintain this position as long as he/she can withstand (3 times over a period of 4 weeks, 10 min each time).

In case of too wide of a mouth opening, or excessive mobility of the jaw and mandible deviation during opening (with excluded suspicion of subluxation), the exercises are limited, and straightening of the opening pathway are recommended. The exercise involves opening the mouth with the tip of the tongue touching the palate (usually near the A-H line) in front of a mirror, along the straight line. It is recommended to maintain the contraction of the tongue muscles for two seconds during mouth opening. The exercises should be repeated 2 to 3 times a day, 15 to 20 repetitions each [3].

### Occlusal splint therapy

To achieve the proper relation of the jaw, centric relation (CR) should be restored. It is easily performed by occlusal splints. An occlusal appliance is any removable artificial occlusal surface used for diagnosis or therapy affecting the relationship of the mandible to the maxillae. Occlusal appliances may be used for occlusal stabilization, for the treatment of temporomandibular disorders, or for the prevention of dentition wear [31]. Occlusal splints are used in a vast majority of patients with TMDs to restore the static and dynamic symmetry of the stomatognathic system. Most commonly, they are used in cases with disc displacement [3, 32, 33]. The splints are fabricated individually by an experienced team consisting of a dentist and technician.

One of the most popular occlusal splints is the Michigan-type bite splint, precisely described by Ramfjord and Ash Jr [34]. This splint could be used in both dental arches, but preferably in the maxilla. The mandibular splint is used when the posterior area is missing teeth in the mandible and unwanted tooth movement must be avoided. The main purpose of this device is to disengage the occlusion, place the condyle in the centric position, relax the masticatory muscles and prevent further tooth wear due to nocturnal parafunctional activity. The main features of this splint are freedom in centric and canine guidance.

It is important to note that the relation of the maxillary and mandibular arches may differ after the treatment when compared to the initial state, especially when partial coverage splints are used [32, 35]. After the replacement of the mandible, the condyles are replaced, and consequently, the mandible is positioned properly and the pain is reduced [32].

Walczynska-Dragon and Baron [10] have proven that occlusal splint therapy using the SVED (Sagittal Vertical Extrusion Device) appliance decreases not only aches in the head and all parts of the spine but also disc displacements within 3 weeks of treatment. The next decrease in frequency of unwanted, unfavorable symptoms was observed after 3 months of treatment with splints. When properly performed, these splints also unblock a limited mouth opening.

Research performed by Lee et al. [36] in a group of 59 patients with somatic TMJ dysfunction showed that intraoral appliance could improve cervical spine alignment and alleviate symptom severity.

The occlusal splints are also used in the initial phase of treatment in patients with mouth overclosure caused by a pathologic deep bite. Before the prosthetic rehabilitation of the severe tooth wear, one should remember that initially, splint therapy should be applied to adapt the stomatognathic system to the new occlusion [37]. A classification of the occlusal appliances with activities and recommendations is presented in Table 2.

**Table 2:** Classification of occlusal splints according to Freesmeyer et al. [28]

Type of occlusal appliances	Activity	Recommendations
Reflex appliances e.g., Interceptor, Anterior Plateau, NTI-tss	Prevent habitual tooth contact and thus prevent gnashing and clenching temporarily, which positively influences the resultant tooth and muscle complaints.	Indicated for acute symptoms that can be attributed to an overloading of the involved tissue (short-term appliances).
Stabilization appliances e.g., Michigan type splint	Create ideal occlusion, synchronous tooth contact in a centric condyle position in static occlusion and an anterior tooth position with disclusion in the lateral teeth region in dynamic occlusion.	Can be used on a short-term and long-term basis, for acute or chronic symptoms and also in psychological and physiological overloading reactions.
Repositioning appliances e.g., Anterior repositioning splint, Farrar type splint, Gelb type splint	The temporomandibular joint or joints is/are set in a therapeutic position by the splint to support healing and to maintain a symptom-free joint posture.	Used for the treatment of temporomandibular joint diseases such as anterior disc displacement with and without reduction, temporomandibular joint compression, retral displacement of the condyle and osteoarthritis. Can be used as a short-term or long-term therapy.

Beside occlusal splint therapy subsequently selective grinding of the teeth to restore physiological and/ or proper and /or balanced occlusal support is required in some cases [27].

### Massage therapy

Myofascial pain is a common symptom of TMD – it is often associated with the clenching of teeth, grinding and stress. TMD myofascial pain occurs in 31 to 76 % of the population [38–41]; it can be relieved by massage therapy, which leads to re-establishing the proper flexibility and muscular length and relieves pain. The massage therapy for TMD might be divided into effleurage, kneading, friction, stretching and petrissage, leading to the permanent adaptation of the muscles. The types of massage and their influence to the surrounding tissues are presented in Table 3 [26, 42]. Massage reduces tissue swelling as well as pain in TMD patients [21, 41, 43]. The pressure used during massage must not be too intense and should increase over time at each therapeutic session because therapy performed too strongly may lead to increased muscular tonus [44]. Massages should be performed twice a week, with a minimum of 30 min for each session. It takes at least 8 therapeutic sessions to receive true relief [23]. In addition to the local influence, massage leads to the relaxation of the entire body and reduces stress, thus improving the patient’s mood [45, 46]; it reduces tension headaches and muscle aches, restores equilibrium between the masseter tension, and improves mastication [26]. The physiotherapist may also recommend heating or cooling of the affected muscles [28].

**Table 3:** Massage procedures in myofascial TMD pain management [26]

Type of movement in massage procedure	Manner of performing	Result
Effleurage, Kneading	Soothing, stroking, circular movements of skin and underlying tissues (performed at the end or beginning of therapeutic session)	Warming up the muscles, providing blood and lymph flow, increasing blood level in the massaged tissues (improved blood flow in small vessels)
Friction	Pressure of fingertips in trigger points therapy; the pressure is increased in particular, sensitive points until the release	Remodeling tissues locally (reconstruction of muscular microstructure); effective in short-term pain relief (activates pain-gate mechanism)
Stretching (“petrissage”)	Rolling of the muscles	Increasing the range of movement and pain relief, decreasing muscle contraction

To restore the accurate function of the TMJ, changes in daily habits is important. The change of food consistency (eating softer foods), applying cold or heat, and avoiding extreme movements of the mandible (chewing gum, wide yawning or loud singing) might be enough to decrease TMD symptoms [47]. In this situation, counselling, behavioral therapy and stress management should also be applied to decrease muscle hypertension and bad habits [25].

### Manual therapy

Manual therapy is similar to massage therapy, but the procedure performed by the physiotherapist is different; it refers to stimulation of the so-called “trigger points”. There are two main methods of treatment by applying manual therapy: mobilization and the muscle energy technique.

The mobilization technique is most commonly used in disc displacements; it involves repeated traction or sliding movements at a slow speed and with increasing amplitude. The desirable effect is to increase the limited range of motion within the joint and reduce pain. The movements are carried out perpendicularly or parallel to the plane of the treated joint, oscillating, and typically repeated 8 to 10 times in 3 sets. The procedure is performed in a seated position with the patient’s head stabilized on the chest of the physiotherapist who holds the patient’s head and mobilizes the mandible with one hand. Traction consists of 3 stages: relaxation (abolition of forces acting on joint), tension (remotion of the articular area) and stretch (increase in remotion of articular area) [48, 49].

The muscle energy technique (MET) is used when limited movements of the mandible are observed and caused by soft tissue (muscles and connective tissue) damage. The treatment involves repeating 3 phases: the first phase is making a movement that is possible due to limited tissue elasticity; in the second phase, the patient slightly tightens the muscles trying to make a move in the opposite direction of the force created by the physiotherapist and should last approximately 10 s; in the last phase, the patient relaxes the muscles. The technique can be performed both in a seated or lying position [50, 51].

### Other physiotherapeutic techniques

Physiotherapy involves many techniques of treatment. The most common massage and manual therapies were previously described, but for TMD treatment, also other techniques are used. Among them, biofeedback, lamp exposure, iontophoresis, ultrasound and transcutaneous electrical nerve stimulation (TENS) are used.

The purpose of biofeedback is to stimulate the muscles to work properly and achieve maximal relaxation of the muscles in a short period of time. The therapy involves electromyography to train the adequate neuromuscular tension of the patient and develops the ability to alter a physiological response. The surface electrodes are placed on the muscles (typically masseter) uni- or bilaterally; other muscles (e.g., anterior temporalis) may also be included. SEMG biofeedback may include muscle tension discrimination. The treatment protocol involves teaching the patient how to open their mouth properly to strengthen the tension of the tongue and protrude the mandible. Only after this are the electrodes applied in line with the muscle fibers (usually upon the midsubstance of the masseter muscle belly). The measurements of the minimal muscular tension are performed when the patient rests with all their muscles relaxed; this is used as a reference in the follow-up. Observing the movements and muscular tonus the patient exercises help to restore the appropriate muscular activity [52–54].

Transcutaneous Electrical Nerve Stimulation (TENS) is another well-known method of pain relief for TMDs. The method is based on electrical stimulation of pain areas via surface electrodes and is considered safe and non-invasive. TENS helps to relieve chronic and acute pain in joint and/or muscle disorders. Unfortunately, due to the small number of studies (especially randomized trials), TENS cannot yet be considered a standard treatment for TMDs, as its effectiveness is still uncertain [55]. In addition to the therapeutic value of electric potential, a tool called electromyography (EMG) is used for establishing muscular function and is the most reliable and objective technique [56].

For pain release, especially in subacute arthropathies and inflammatory rheumatic diseases, heat treatment is applied; it alleviates strong pain, although the result is typically short-therm. Heat is supplied either by means of Solux lamps (ca. 15 min from 20 cm distance) or through a thermophor filled with water at a temperature of 158 to 176 °F (70 to 80 °C) and wrapped with a towel. Other recommendations to decrease pain are sulfur and iodide baths. Cryotherapy is another form of temperature related therapy but applies cold instead of heat. Cold packs, cold spray or air, and ice compresses are used as analgesic agents. The application of cold is used immediately prior to kinesiotherapy and helps fight muscle hypertension and tendinopathies as well as rheumatic diseases. One should remember that there is a high risk of frostbite (skin damage due to low temperature) with this form of therapy. The cold compresses should be applied for 10–15 min. Cryotherapy leads to the attenuation of pain, reduces stiffness in the TMJ and increases mandibular mobility [57].

A new method of rehabilitation with the aim of TMJ stabilization and increased jaw stability is taping, or Kinesio Taping (KT). KT also decreases drooling and provides mouth closure. To increase jaw stability, one piece of tape in a “Y”-shape cut should be prepared and placed proximal to the joint; the superior tail is shorter than the inferior tail. The superior tail should be applied diagonally along the upper jaw and directed towards the lower cheek with “paper-off” tension. The tape width should be 1.5 to 2 inches (3.8 to 5 cm). The mandible cannot subluxate at the movement. To decrease the hypermobility of the joint and release TMJ pain, two tape pieces (1 inch wide and 2 inches long each) should be placed diagonally to each other over the joint, forming an “X”. To improve jaw stability, tape is usually applied to both sides. The balance in head position and body posture usually leads to a decrease in hypertension of not only the masticatory muscles but also the neck, arms and spine [58, 59]. The method is quite new but has become increasingly popular [60, 61]. The special therapeutic tape adheres to the skin with adequate flexibility and consists of a polymer elastic strand wrapped by 100 % cotton fibers. The tape allows for a normalization of muscle tone and increases the process of self-healing. KT stimulates an endogenous analgesic system and changes the subjective feelings of the patient. Alignment of muscular tone is possible by improving proprioception. KT could be applied for myofascial pain therapy in a range of masticatory muscles, especially the masseters. The clinical technique has been described by Kase et al. [62].

Ultrasound therapy is one of the efficacious methods for pain reduction, decrease in muscular tonus and improving the function of the muscles. It consists of three types of signals: constant waves, sound impulses and ultrasound combined with stimulation current, which is found to be most effective. The procedure is performed 6–12 times, every 1–2 days, 6–8 min each. The impulses should be applied at 0.5–0.7 W/cm in the case of devices with constant waves, and 0.6–0.9 W/m in the case when sound impulses (50 or 100 Hz) are emitted [57].

There are few rarely used methods of TMD management. Among them are iontophoresis with different medications (e.g., nonsteroidal anti-inflammatory drugs, steroids and analgesics), especially in patients with concurrent temporomandibular joint disc displacement without reduction and capsulitis [63]. As the data show, pain release is not observed, but patients present with a wider opening of the mouth than when analgesics alone are used [63]. Inflammatory processes may be healed with a laser light that is used at a wavelength of 904 nm and a frequency of 700 Hz at 30 mm depth into the skin. This method had gained popularity [57].

## Pharmacotherapy and minimally invasive and invasive procedures

### Oral and injectable pharmacotherapy

Pharmacotherapy for TMD is not commonly used. It is only used when other somatic symptoms, such as sleep disorders, chronic pain, arthralgias, inflammatory diseases, myalgias or neuropathies are associated with TMD [28]. As TMD may manifest from different systemic diseases (e.g., arthritis, inflammatory bowel diseases, Parkinson disease), it is important to diagnose the patient properly and implement treatment for the underlying disease, especially when depression is a suspected diagnosis [47, 64]. One has to remember that pharmacotherapy has its goal in decreasing pain and inflammation within the joint and/or muscles. This therapy improves function and inhibits the progression of the disease [65]. Pharmacotherapy can be considered as a complementary therapy rather than a treatment itself. The exceptions are systemic diseases with TMJ involvement [57].

For TMD release, the most commonly used medications are myorelaxants, nonsteroidal anti-inflammatory drugs (NSAIDs), analgesics, tricyclic antidepressants, benzodiazepines and corticosteroids [28]. The first medication of choice for moderate pain relief is acetaminophen (average daily dose of 325–1000 mg). NSAIDs and analgesics help to relieve pain (including radiating pain) in the head, jaw muscles, face, neck or shoulders. A high efficiency of TMD pain relief is shown with ibuprofen* and meloxicam** (average daily dose of 400–800 mg* and 7.5–15 mg**). In this particular situation, pharmacotherapy is considered a supportive therapy that supplements other therapies. Used by itself, pharmacotherapy is considered for palliative therapy [48]. NSAIDs decrease pain and stop the inflammatory process [64].

Muscle relaxants (baclofen, tizanidin, cyclobenzaprine), opiates (morphine), anticonvulsants (e.g., gabapentin), ketamine, and TCA (e.g., amitriptyline) have also been used clinically for TMJ management, but there is no evidence for their efficacy [65, 66]. To achieve the myorelaxation effect with low CNS impact, metaxolone is recommended (average daily dose of 800 mg).

In specific cases, medications should be used admittedly. During acute spasms (sudden muscular contraction and painful shortening that is maintained over time), anesthetics are advised to block the pain and allow therapeutic stretching. Usually, the analgesic blockage with an infiltration of 1 ml of 2 % lidocaine (without vasoconstrictor) in the involved muscle is applied. A complementary therapy may include dypirone 500 mg (also in association with a myorelaxant, such as orfendrine, if necessary) 3 times a day, for 2 days [46]. In this situation, 90 % of cases require analgesic therapy [65].

In myositis and other inflammatory disorders, the most appropriate strategy is the administration of one dose of corticosteroid intramuscularly. Another approach is the injection of an analgesic or anti-inflammatory agent. The most common injections contain corticosteroids (with anti-inflammatory action) or hyaluronic acid [67]. In animal models, the use of an inhibitor selective for the inducible COX-2 enzyme may attenuate the neurogenic component of inflammation [47]. COX enzymes are blocked by NSAIDs. Unfortunately, those medications have a high risk of adverse side effects, which may include exacerbation of hypertension or gastrointestinal upset that may lead to ulcerations. COX-2-selective NSAIDs (eg. Celecoxib, Meloxicam) which have less side effects, are not found to be better for the treatment of TMD. There is a hope that lotions containing NSAIDs will not have as many side effects and will have a positive impact on relieving pain [65].

In chronic facial pain, aside from pain relievers, antidepressants should be used as a supplementary treatment [47]. Antidepressants may be used for chronic pain as a primary analgesic. These medications manage headaches and neuropathic pain, reducing the feeling of depression caused by pain and improving sleep quality [65].

It had been proven that NSAIDs relieve pain in patients who suffer from arthritis. In this situation, diclofenac at a maximum dose of 50 mg orally 3 times daily or naproxen sodium 500 mg twice a day are recommended, as they improve pain in more than half of the patients [65]. It had been shown that the use of antibiotics, such as doxycycline or other tetracyclines, could help prevent condylar resorption. Regardless of their antibiotic activity, antibiotics inhibit matrix metalloproteinases (MMPs), whose levels are elevated in inflammatory processes involving TMJ [67]. Doxycycline is also a medication of choice in patients who undergo orthognathic surgery to avoid the resorption process [68].

For anxiety treatment and stress relieve, benzodiazepine (eg. Diazepam 5 mg, Lorazepam 1 mg or Alprazolam 0.5 mg) for 5–10 days should be prescribed [46].

Clinical investigations by Bakke et al. [69] and Emara et al. [70] confirm the possibility of applying botulinum toxin type A (BTX-A) for the treatment of disc displacements using injections in the lateral pterygoid muscles. BTX-A decreases myofascial pain and symptoms in the bruxers by reducing muscle tension [71].

Botulin is a biologic neuromuscular blocking agent that works as a muscle relaxant and therefore relieves pain in the head and neck; it also decreases neuromuscular tonus and bruxing at night. Hypertrophic masseter muscles activity is also reduced. Due to the large scope of BTX-A, it can be used in various temporomandibular disorders, such as bruxism, oromandibular dystonia, myofascial pain (also including TMJ involvement), trismus, hypermobility, masseter or temporalis hypertrophy, headaches and neck pain [72, 73].

### Acupuncture

A common method frequently used in Asian countries is a needle puncture, also known as acupuncture. This method is also gaining popularity in western countries. Acupuncture originated in China over 3,000 years ago. A skilled acupuncturist restores whole body balance and the flow of energy within it (called *Qi*) to relieve a patient’s pain and to improve the inflammatory process within the joint and decrease hypertension. The method is more successful in patients who change their dietary habits (soft food, avoidance of chewing gum, less saturated fats, coffee and fried foods in the diet). Interestingly, acupuncture is very successful in long-term follow-ups (18–20 years). There are several recommended acupuncture points (e.g., SI-18, GV-20, GB-20, ST-6, ST-7, BL-10 and LI-4) that should be “triggered” weekly, 30 min per session. Needles are inserted within the pain area and around the ear and jaw. In some cases, needles near elbows, knees and the big toe are inserted to relieve pain and inflammatory process within the TMJ. It is recommended to complete 6 sessions of acupuncture treatment, but chronic disorders may require more. Often, acupuncture should be associated with pharmacotherapy [66, 74–76].

A modern approach of needle puncture is based on the findings of trigger points in painful muscles [77]. Dry needles are inserted at the trigger points, or taut bands, which are not related to the meridian or Chi points, are placed according to traditional Chinese acupuncture practices [78, 79]. Biochemical differences have been found between healthy muscle fibers, and active and latent trigger points [80]. Therefore, needle puncture at trigger points actually change the biochemical environment of the painful muscles of TMD patients.

### Drug therapy and alternatives in rheumatoid disorders

In rheumatoid disorders, the TMJ is usually only one of the joints (or only one of the organs) involved in the disease process. The pharmacologic treatment in this case plays a crucial role and is not only an adjuvant therapy. In those cases, pharmacologic treatment refers to the whole systemic disease and not only to the TMD.

Among patients with juvenile idiopathic arthritis (JIA), joint involvement may be accompanied by periodontal disorders and gingivitis; it usually shows no relation in higher incidences of the caries process. TMD in this disorder are confirmed by the Ai Helkimo and Di Helkimo indexes, which show that disorders within this joint are reported both objectively and subjectively [81]. Patients with JIA or RA (rheumatoid arthritis) are believed to suffer from TMD in 1 to 25 % of cases, but up to 75 % prevalence might be observed. Arthritis may be asymptomatic but might be associated with TMJ pain, especially during movement. The disorders may include condylary damage and synovitis. The untreated process may lead to mandibular growth disturbances, leading to laterogenia, malocclusions and micrognathia. The joint involvement would, in this case, impact the treatment decisions. In pharmacotherapy, systemic methotrexate and/or TNF inhibitors are used. Additionally, corticosteroids might be successful for modifying the course of the disease. Splint therapies and functional orthodontic appliances might still be used but are adjuvant to the pharmacologic treatment. The medications themselves may reduce the inflammatory process within the joint [82–85].

### Surgical procedures

The arthrocentesis that involves draining the joint with a therapeutic substance reduces the inflammatory process, evacuates inflammatory exudate, releases the disc, breaks up adhesions, eliminates pain, and improves joint mobility; this should be performed with the mouth wide open and a protruded mandible [80, 85]. Two needles are used to puncture the joint space to restore normal maximal mouth opening and functioning. This technique has limitations due to low tolerability and difficulties in performing the procedure; therefore, single needle arthrocentesis has become more popular [86]. Randomized controlled trial carried out by Vos et al. [87] tried to determine the effectiveness of arthrocentesis compared to conservative treatment as initial treatment with regard to temporomandibular joint pain and mandibular movement. They showed that arthrocentesis reduces pain and functional impairment more rapidly compared to conservative treatment but in long term observations the effectivnes of both treatment modalities achieved comparable outcomes.

The method of intra-articular injections of platelet-rich plasma (PRP) to patients with persistent pain related to severe temporomandibular joint dysfunction described by Pihut et al. [88] seems to be a valid procedure for decreasing TMD pain.

In the most severe cases in who TMJ is too severely damaged by the inflammatory process to be cured in a conservative way, implants are used to replace the TMJ. Examples include the Christensen system, the TMJ Concepts system and the Lorenz (BMF) system. Ciocca et al. [89] showed the regenerative properties of mesenchymal stem cells and CAD-CAM-customized pure and porous hydroxyapatite scaffolds to replace the temporomandibular joint condyle. Previously mentioned articles and other papers have confirmed that tissue engineering and stem cells therapy seem to be a promising alternative to the traditional procedures for the management of pain associated with degenerative TMJ disease [90, 91].

The main indication for TMJ replacement is pain relief and functional improvement in arthritis (osteoarthritis, psoriatic, rheumatoid arthritis and ankylosing spondylitis). The other situations where the TMJ needs replacement are ankylosis, damage by trauma and complications after earlier joint replacement [92, 93]. In a a case of severe malocclusion, dentofacial anomalies and unilateral condylar hyperplasia or hypoplasia complicated by TMJ dysfunction the surgical procedures combined with orthodontic treatment should be considered [94, 95].

## Conclusions

**Fig. 1:**
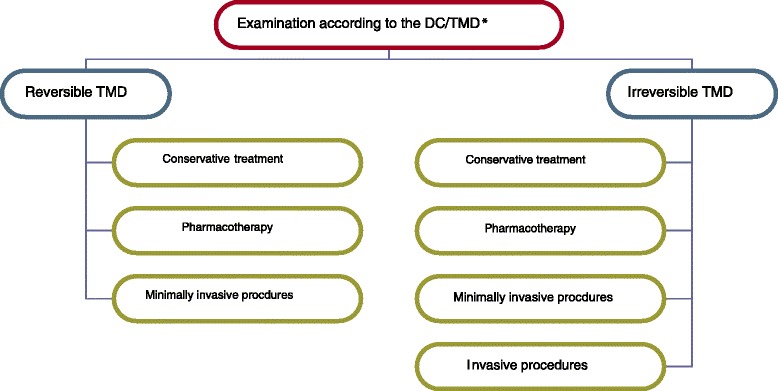
Treatment algorithm for the management of TMD-related pain (*The Diagnostic Criteria for Temporomandibular Disorders by Schiffman et al. [1])

Due to the diverse causes of these disorders, TMD pain management requires various methods of treatment that are conformable to the origin of the dysfunction (Table 4). The authors concluded that conservative treatment including counselling, exercises, occlusal splint therapy, massage, manual therapy and others should be considered as the first choice treatment for TMD pain because of their low risk of side effects. In cases of severe acute or chronic pain resulting from serious disorders, inflammation and/or degeneration pharmacotherapy, minimally invasive and invasive procedures should be included (Fig. 1).

**Table 4:** Reported treatment modalities related to the selected disease entities associated with temporomandibular disorders

Type of the disease entity acc. ICD-10^a^	Treatment modalities	Type and year of the selected confirming article	Authors
Pain disorders:			
1. Myalgia, Myofascial pain (M79.1)	Counselling; Occlusal splint therapy; Massage; Manual therapy; Other physiotherapeutic techniques; Oral and injectable drug therapy	Review (1994)	Ramfjord et al. [34]
		Original (2004)	Magnusson et al. [35]
		Original (2009)	Hamata et al. [32]
2. Arthralgia (M26.62)		Review (2012)	Miernik et al. [26]
3. Headache attributed to TMD (G44.89)		Original (2003)	Hilbert et al. [46]
		Review (2007)	Smith [44]
4. Tension-type headache (G44.2)		Review (2010)	Cairns [65]
		Original (2008)	Guarda-Nardini et al. [71]
Joint disorders:			
1. Disc displacement (M26.63)	Counselling; Therapeutic exercises; Occlusal splint therapy; Massage; Manual therapy; Other physiotherapeutic techniques; Oral and injectable drug therapy; Minimally invasive and invasive surgical procedures	Original (2013)	Bae et al. [30]
		Review (1994)	Ramfjord et al. [34]
2. Degenerative joint disease (M19.91)		Original (2004)	Magnusson et al. [35]
3. Subluxation (S03.0XXA)		Original (2009)	Hamata et al. [32]
4. Derangement of TMJ (K07.6)		Review (2012)	Miernik et al. [26]
5. Arthritis of TMJ (K07.6)		Original (2003)	Hilbert et al. [46]
6. Injuries of TMJ (S03.0-dislocation; S01.4-open wound; S02.6-fracture)		Review (2007)	Smith [44]
		Original (1996)	Schiffman et al. [63]
		Review (2010)	Cairns [65]
		Original (2007)	Gunson et al. [67]
		Original (2013)	Emara et al. [70]
		Original (2014)	Vos et al. [87]
		Original (2013)	Sidebottom et al. [93]
Bruxism:			
1. Psychogenic (F45.8)	Counselling; Psychotherapy; Occlusal splint therapy; Massage; Other physiotherapeutic techniques; Oral and injectable drug therapy	Review (1994)	Ramfjord et al. [34]
		Original (2004)	Magnusson et al. [35]
2. Sleep related (G47.63)		Original (2009)	Hamata et al. [32]
		Review (2012)	Miernik et al. [26]
		Original (2003)	Hilbert et al. [46]
		Review (2006)	Medlicott et al. [51]
		Review (2010)	Cairns [65]
		Original (2008)	Guarda-Nardini et al. [71]
Excessive attrition of teeth (K03.0)	Counselling; Occlusal splint therapy; Prosthodontic rehabilitation	Review (1994)	Ramfjord et al. [34]
		Original (2004)	Magnusson et al. [35]
		Review (2011)	Johansson et al. [37]
Anomalies of dental arch relationship (K07.2); Dentofacial anomalies (K07.0; K07.1); Unilateral condylar hyperplasia or hypoplasia (K10.8)	Counselling; Occlusal splint therapy; Prosthodontic rehabilitation; Orthodontic therapy; Invasive surgical procedures	Review (1994)	Ramfjord et al. [34]
		Original (2004)	Magnusson et al. [35]
		Review (2011)	Johansson et al. [37]
		Original (1997)	Gerbino et al. [94]
		Original (2013)	Abrahamsson et al. [95]

^a^ *ICD-10* International Classification of Diseases 10^th^ Revision

## References

[1] Schiffman E, Ohrbach R, Truelove E, Look J, Anderson G, Goulet JP (2014). Diagnostic Criteria for Temporomandibular Disorders (DC/TMD) for Clinical and Research Applications: recommendations of the International RDC/TMD Consortium Network and Orofacial Pain Special Interest Group. J Oral Facial Pain Headache.

[2] Kobs G, Bernhardt O, Kocher T, Meyer G (2005). Oral parafunctions and positive clinical examination findings. Baltic Dent Maxillofac J.

[3] Kijak E, Lietz-Kijak E, Śliwiński Z, Frączak B (2013). Muscle activity in the course of rehabilitation of masticatory motor system functional disorders. Postepy Hig Med Dosw.

[4] Liu F, Steinkeler A (2013). Epidemiology, diagnosis, and treatment of temporomandibular disorders. Dent Clin North Am.

[5] Miettinen O, Lahti S, Sipilä K (2012). Psychosocial aspects of temporomandibular disorders and oral health-related quality-of-life. Acta Odontol Scand.

[6] Manfredini D, Borella L, Favero L, Ferronato G, Guarda-Nardini L (2010). Chronic pain severity and depression/somatization levels in TMD patients. Int J Prosthodont.

[7] Bono AE, Learreta JA, Rodriguez G, Marcos JC (2014). Stomatognathic system involvement in rheumatoid arthritis patients. CRANIO.

[8] Fernandes G, Gonçalves DA, de Siqueira JT, Camparis CM (2013). Painful temporomandibular disorders, self reported tinnitus, and depression are highly associated. Arq Neuropsiquiatr.

[9] Calixtre LB, Grüninger BL, Chaves TC, Oliveira AB (2014). Is there an association between anxiety/depression and temporomandibular disorders in college students?. J Appl Oral Sci.

[10] Walczynska-Dragon K, Baron S (2011). The biomechanical and functional relationship between temporomandibular disfunction and cervical spine pain. Acta Bioeng Biomech.

[11] Sipilä K, Suominen AL, Alanen P, Heliövaara M, Tiittanen P, Könönen M (2011). Association of clinical findings of temporomandibular disorders (TMD) with self-reported musculoskeletal pains. Eur J Pain.

[12] Gonçalves DA, Bigal ME, Jales LC, Camparis CM, Speciali JG (2010). Headache and symptoms of temporomandibular disorder: an epidemiological study. Headache.

[13] Zakrzewska JM (2013). Multi-dimensionality of chronic pain of the oral cavity and face. J Headache Pain.

[14] Fujita Y, Motegi E, Nomura M, Kawamura S, Yamaguchi D, Yamaguchi H (2003). Oral habits of temporomandibular disorder patients with malocclusion. Bull Tokyo Dent Coll.

[15] de Barbosa TS, Miyakoda LS, de Pocztaruk RL, Rocha CP, Gaviao MB (2008). Temporomandibular disorders and bruxism in childhood and adolescence: review of the literature. Int J Pediatr Otorhinolaryngol.

[16] Kibana Y, Ishijima T, Hirai T (2002). Occlusal support and head posture. J Oral Rehabil.

[17] Okeson JP, de Leeuw R (2011). Differential diagnosis of temporomandibular disorders and other orofacial pain disorders. Dent Clin North Am.

[18] Sokalska J, Wieckiewicz W, Zenczak-Wieckiewicz D (2006). Influence of habit of chewing gum on condition of stomatognathic system. Dent Med Probl.

[19] Cuccia A, Cardonna C (2009). The relationship between stomatognathic system and body posture. Clinics.

[20] Loster JE, Wieczorek A (2014). An assessment of the effectiveness of treatment for temporomandibular joint dysfunctions. Dent Med Probl.

[21] Shamim T (2014). The psychosomatic disorders pertaining to dental practice with reviesed working type classification. Korean J Pain.

[22] Grippo JO (1991). Abfractions: a new classification of hard tissue lesions of teeth. J Esthet Restor Dent.

[23] Grippo JO, Simring M, Schreiner S (2004). Attricion, abrasion, corrosion and abfraction revisited. A new perspective on toth surface lesions. JADA.

[24] Grippo JO, Simring M, Coleman TA (2012). Abfraction, abrasion, biocorrosion, and the enigma of noncarous cervical lesions: a 20-year perspective. J Esthet Restor Dent.

[25] Chandola HC, Chakraborty A (2009). Fibrynomyalgia and myofascial pain syndrome – a dilemma. Indian J Anaesth.

[26] Miernik M, Wieckiewicz M, Paradowska A, Wieckiewicz W (2012). Massage therapy in myofascial TMD pain management. Adv Clin Exp Med.

[27] Bhat S (2010). Etiology of temporomandibular disorders: the journey so far. Int Dent SA.

[28] Freesmeyer WB, Fussnegger MR, Ahlers MO (2005). Diagnostic and therapeutic-restorative procedures for masticatory dysfunctions. GMS Curr Top Otorhinolaryngol Head Neck Surg.

[29] Robson FC (2001). The clinical evaluation of posture: relationship of the jaw and posture. CRANIO.

[30] Bae Y, Park Y (2013). The Effect of Relaxation Exercises for the Masticator Muscles on Temporomandibular Joint Dysfunction (TMD). J Phys Ther Sci.

[31] The Academy of Prosthodontics (2005). The glossary of prosthodontics terms 8th edition. J Prosthet Dent.

[32] Hamata MM, Zuim PRJ, Garcia AR (2009). Comparative evaluation of the efficacy of occlusal splints fabricated in centric relation or maximum intercuspidation in centric relation or maximum intercuspidation in temporomandibular disorders patients. J Appl Sci.

[33] Ash MM, Ramfjord SP (1998). Reflections on the Michigan splint and other intraocclusal devices. J Mich Dent Assoc.

[34] Ramfjord SP, Ash MM (1994). Reflections on the Michigan occlusal splint. J Oral Rehabil.

[35] Magnusson T, Adiels AM, Nilsson HL, Helkimo M (2004). Treatment effect on signs and symptoms of temporomandibular disorders--comparison between stabilisation splint and a new type of splint (NTI). A pilot study. Swed Dent J.

[36] Lee YJ, Lee JK, Jung SC, Lee H, Yin CS, Lee YJ (2013) Case series of an intraoral balancing appliance therapy on subjective symptom severity and cervical spine alignment. eCAM. 1–7. doi:10.1155/2013/181769 10.1155/2013/181769 PMC3713604 23935655

[37] Johansson A, Omar R, Carlsson GE (2011). Bruxism and prosthetic treatment: a critical review. J Prosthodont Res.

[38] LeResche L, Mancl LA, Drangsholt MT, Huang G, Von Korff M (2007). Predictors of onset of facial pain and temporomandibular disorders in early adolescence. Pain.

[39] Glaros AG, Williams K, Lausten L (2005). The role of parafunctions, emotions and stress in predicting facial pain. JADA.

[40] Van Selms MK, Lobbezoo F, Visscher CM, Naeije M (2008). Myofascial temporomandibular disorder pain, parafunctions and psychological stress. J Oral Rehabil.

[41] Yap AU, Dworkin SF, Chua EK, List T, Tan KB, Tan HH (2003). Prevalence of temporomandibular disorder subtypes, psychologic distress and psychosocial dysfunction in Asian patients. J Orofac Pain.

[42] List T, Dworkin SF (1996). Comparing TMD diagnoses and clinical findings at Swedish and US TMD centers using research diagnostic criteria for temporomandibular disorders. J Orofac Pain.

[43] Goats GC (1994). Massage – the scientific basis of an ancient art: part 1. The techniques. Br J Sports Med.

[44] Smith AR (2007). Manual therapy: the historical, current, and future role in the treatment of pain. Sci World J.

[45] Roberts L (2011). Effects of patterns of pressure application on resting electromyography during massage. Int J Ther Massage Bodywork.

[46] Hilbert JE, Sforzo GA, Swensen T (2003). The effects of massage on delayed onset muscle soreness. Br J Sports Med.

[47] Hatayama T, Kitamura S, Tamura C, Nagano M, Ohnuki K (2008). The facial massage reduced anxiety and negative mood status, and increased sympathetic nervous activity. Biomed Res.

[48] de Andrade ED, Rizzatti-Barbosa CM, Pimenta Pinheiro ML (2004). Pharmacological guidelines for managing temporomandibular disorders. Braz J Oral Sci.

[49] Yabe T, Tsuda T, Hirose S, Ozawa T, Kawai K (2014). Treatment of the acute temporomandibular joint dislocation using manipulation technique for disk displacement. J Craniofac Surg.

[50] Alves BM, Macedo CR, Januzzi E, Grossmann E, Atallah AN, Peccin S (2013). Mandibular manipulation for the treatment of temporomandibular disorder. J Craniofac Surg.

[51] Medlicott MS, Harris SR (2006). Temoporomandibular disorder training, and biofeedback in the management of relaxation exercise, manual therapy, electrotherapy, a systematic review of the effectiveness. Phys Ther.

[52] Rajadurai V (2011). The effect of the muscle energy technique on temporomandibular joint disfunction. A randomized clinical trial. Asian J Sci Res.

[53] Crider A, Glaros AG, Gavirtz RN (2005). Effccacy of biofeedback-based treatments for temporomandibular disorders. Appl Psychophysiol Biofeedback.

[54] Crider AB, Glaros AG (1999). A meta-analysis of EMG-biofeedback treatment of temporomandibular disorders. J Orofac Pain.

[55] Canavan P, Capurso J (2007) Electromyography in physical therapy and dentistry. Protocol for use of EMG and tactile biofeedback in treatment of temporomandibular disorders and myofascial pain. The Biofeedback Federation of Europe Clinical Protocols (Accessed March 6, 2007, at https://bfe.org/new/news/protocols/ Protocol 6 March 2007.pdf)

[56] Moger G, Sashikanth MC, Sunil MK, Shambulingappa P (2011). Transcutaneous electrical nerve stimulation therapy in temoporomandibular disorder: a clinical study. JIAOMR.

[57] Wozniak K, Piatkowska D, Lipski M, Mehr K (2013). Surface electromyography in orthodontics – a literature review. Med Sci Monit.

[58] Kogut G, Kwolek A (2006). Functional disturbances of the masticatory apparatus – diagnosis and treatment. Med Rehabil.

[59] Wozniak K, Piatkowska D, Lipski M (2012). The influence of natural head position on the assessment of facial morphology. Adv Clin Exp Med.

[60] Mostafavifar M, Wertz J, Borchers J (2012). A systemic review of the efectiveness of Kinesio taping for musculoskeletal injury. Phys Sportsmed.

[61] Kaya E, Zinnuroglu M, Tugcu I (2011). Kinesio taping compared to physical therapy modalities for the treatment shoulder impingement syndrome. Clin Rheumatol.

[62] Kase K, Wallis J, Kase T (2013). Clinical therapeutic applications of the Kinesio Taping method.

[63] Schiffman EL, Brown BL, Lindgren BR (1996). Temporomandibular joint iontophoresis: a double-blind randomized clinical trial. J Orofac Pain.

[64] Wieckiewicz M, Paradowska A, Kawala B, Wieckiewicz W (2011). SAPHO syndrome as a possibile cause of masticatory system anomaly – a review of the literatue. Adv Clin Exp Med.

[65] Cairns BE (2010). Pathophysiology of TMD pain – basic mechanisms and their implications for pharmacotherapy. J Oral Rehabil.

[66] Shen YF, Goddard G (2007). The short-term effects of acupuncture on myofascial pain patients after clenching. Pain Pract.

[67] Gunson MJ, Arnett GW (2010). Condylar resorption, matrix metalloproteinases, and tetracyclines. RWISOJ.

[68] Heir GM, Haddox DJ, Crandall J, Eliav E, Radford SG, Schwartz A (2011). Appropriate use of pharmacotherapeutic agents by the orofacial pain dentist. J Orofac Pain.

[69] Bakke M, Møller E, Werdelin LM, Dalager T, Kitai N, Kreiborg S (2005). Treatment of severe temporomandibular joint clicking with botulinum toxin in the lateral pterygoid muscle in two cases of anterior disc displacement. Oral Surg Oral Med Oral Pathol Oral Radiol Endod.

[70] Emara AS, Faramawey MI, Hassaan MA, Hakam MM (2013). Botulinum toxin injection for management of temporomandibular joint clicking. Int J Oral Maxillofac Surg.

[71] Guarda-Nardini L, Manfredini D, Salamone M, Salmaso L, Tonello S, Ferronato G (2008). Efficacy of botulinum toxin in treating myofascial pain in bruxers: a controlled placebo pilot study. CRANIO.

[72] Schwarz M, Freund B (2002). Treatment of temporomandibular disorders with botulinum toxine. Clin J Pain.

[73] Ho KY, Tan KH (2007). Botulinum toxin A for myofascial trigger point injection: A qualitative systemic review. Eur J Pain.

[74] Rosted P (2001). Practical recommendations for the use of acupuncture in treatment of temporomandibular disordes based on the outcome of published controlled studies. Oral Dis.

[75] Rosted P, Bundgaard M, Pedersen AM (2006). The use of acupuncture in the treatment of temporomandibular dysfunction – an audit. Acupunct Med.

[76] Bergstrðm I, List T, Magnusson T (2008). A follow-up study of subjective symptoms of temporomandibular disorders in patients who received acupuncture and/or interocclusal appliance therapy 18–20 years earlier. Acta Odontol Scand.

[77] Mense S (2003). The pathogenesis of muscle pain. Curr Pain Headache Rep.

[78] Hong C (1994). Lidocain injection versus dry needling to myofascial trigger points: the importance of local twitch response. Am J Phys Med Rehabil.

[79] Langevin H, Audette JF, Bailey A (2008). Potential role of fascia in chronic musculoskeletal pain. Integrative pain medicine: The science and practice of complementary and alternative medicine in pain management.

[80] Shah JP, Gilliams EA (2008). Uncovering the biochemical milieu of myofascial trigger points using in vitro microdialysis: An application of muscle pain concepts to myofascial pain syndrome. J Bodyw Mov Ther.

[81] Gmyrek-Marciniak A, Kaczmarek U (2012). Oral condition in children and adolescents suffering from juvenile idiopathic arthritis. Dent Med Probl.

[82] Ringold S, Tzaribachev N, Cron RQ (2012). Management of temporomandibular joint arthritis in adult rheumatology practices: a survey of adult rheumatologists. Pediatr Rheumatol.

[83] Habibi S, Ellis J, Strike H, Ramanan AV (2012). Safety and efficacy of US-guided CS injection into temporomandibular joints in children with active JIA. Rheumatol.

[84] Carvalho RT, Braga FS, Brito F, Capelli J, Figueredo CM, Sztainbok FR (2012). Temporomandibular joint alternations and their orofacial complications in patients with juvenile idiopathic arthritis. Rev Bras Reumatol.

[85] De Riu G, Stimolo M, Meloni SM, Soma D, Pisano M, Sembronio S et al. (2013) Arthrocentesis and temporomandibular joint disorders: clinical and radiological results of prospective study. Int J Dent. 1–8. doi:10.1155/2013/790648. 10.1155/2013/790648 PMC3844254 24319462

[86] Singh S, Varghese D (2013). Single puncture arthrocentesis of temporomandibular joint; introducing a novel device: A pilot study. Natl J Maxillofac Surg.

[87] Vos LM, Huddleston Slater JJ, Stegenga B (2014). Arthrocentesis as initial treatment for temporomandibular joint arthropathy: A randomized controlled trial. J Craniomaxillofac Surg.

[88] Pihut M, Szuta M, Ferendiuk E, Zenczak-Wieckiewicz D (2014) Evaluation of pain regression in patients with temporomandibular dysfunction treated by intra-articular platelet-rich plasma injections: a preliminary report. Biomed Res Int. 1–7. doi:10.1155/2014/132369. 10.1155/2014/132369 PMC4137492 25157351

[89] Ciocia L, Donati D, Ragazzini S, Dozza B, Rossi F, Fantini M et al. (2013) Mesenchymal Stem Cells and Platelet Gel Improve Bone Deposition within CAD-CAM Custom-Made Ceramic HA Scaffolds for Condyle Substitution. Biomed Res Int. 1–10. doi:10.1155/2013/549762. 10.1155/2013/549762 PMC3773948 24073409

[90] Abou Neel EA, Chrzanowski W, Salih VM, Kim HW, Knowles JC (2014). Tissue engineering in dentistry. J Dent.

[91] Wu Y, Gong Z, Li J, Meng Q, Fang W, Long X (2014) The Pilot Study of Fibrin with Temporomandibular Joint Derived Synovial Stem Cells in Repairing TMJ Disc Perforation. Biomed Res Int. 1–10. doi:10.1155/2014/454021. 10.1155/2014/454021 PMC4009306 24822210

[92] Speculand B (2009). Current status of replacement of the temporomandibular joint in the United Kingdom. Br J Oral Maxillofac Surg.

[93] Sidebottom AJ, Gruber E (2013). One-year prospective outcome analysis and complications following total replacement of the temporomandibular joint with the TMJ Concepts system. Br J Oral Maxillofac Surg.

[94] Gerbino G, Bianchi SD, Bernardi M, Berrone S (1997). Hyperplasia of the mandibular coronoid process: long-term follow-up after coronoidotomy. J Craniomaxillofac Surg.

[95] Abrahamsson C, Henrikson T, Nilner M, Sunzel B, Bondemark L, Ekberg EC (2013). TMD before and after correction of dentofacial deformities by orthodontic and orthognathic treatment. Int J Oral Maxillofac Surg.

